# Chronic Kidney Disease and Chronic Oral Inflammatory Diseases: A Systematic Review and Meta-Analysis of Periodontitis and Apical Periodontitis

**DOI:** 10.3390/jcm14227947

**Published:** 2025-11-10

**Authors:** Laura López-Sanz, María León-López, Sonia Egido-Moreno, Carlos Segura-Raya, José López-López, Juan J. Segura-Sampedro, Daniel Cabanillas-Balsera, Juan J. Segura-Egea

**Affiliations:** 1 Department of Stomatology (Endodontics Section), School of Dentistry, University of Sevilla, C/Avicena s/n, 41009 Sevilla, Spain; laulopsan@alum.us.es (L.L.-S.); mleon12@us.es (M.L.-L.);; 2 Medicine and Health Sciences Faculty, University of Barcelona, 08007 Barcelona, Spain; soniaegido@ub.edu (S.E.-M.);; 3 Oral Health and Masticatory System Group, IDIBELL (Bellvitge Biomedical Research Institute), University of Barcelona, 08007 Barcelona, Spain; 4 Department of Clinical Medical Sciences, School of Medicine, Universidad San Pablo CEU, 28040 Madrid, Spain; 5 Hospital La Paz Institute for Health Research (IdiPAZ), 28046 Madrid, Spain

**Keywords:** apical periodontitis, chronic kidney disease, oral infections, periodontitis, systematic review, meta-analysis

## Abstract

**Background**: Chronic kidney disease (CKD) has been increasingly associated with oral chronic inflammatory conditions, including periodontitis (PD) and apical periodontitis (AP). Both share common pathophysiological pathways involving systemic inflammation, immune dysregulation, and oxidative stress. This systematic review and meta-analysis aimed to synthesize current evidence on the association between CKD and chronic oral inflammatory diseases. **Methods**: The PRISMA guidelines were followed and the proto-col was registered in PROSPERO: CRD420251167323. A comprehensive electronic search was conducted in PubMed, Scopus, Web of Science, and ProQuest up to September 2025. Observational studies reporting prevalence of chronic oral inflammatory diseases in CKD patients and controls subjects were included. The Newcastle–Ottawa scale was used for assessing risk of bias. Pooled odds ratios (ORs) were calculated using a random-effects model. **Results**: Seven studies published between 2011 and 2025, including 13,139 participants, met the inclusion criteria. CKD patients had significantly higher prevalence of oral inflammatory disease than controls (OR = 4.2; 95% CI = 2.5–7.2; *p* < 0.00001). Heterogeneity was high (I^2^ = 83.0%). Subgroup analysis showed an OR of 4.3 (95% CI = 2.6–7.0; *p* < 0.0001) for AP and 4.3 (95% CI = 2.2–8.7) for PD. The overall risk of bias was moderate, and the certainty of evidence according to GRADE was rated as low. **Conclusions** This systematic review and meta-analysis highlight a potential link between chronic oral inflammatory disease, including both AP and PD, and chronic kidney disease (CKD). However, the certainty of the evidence is low, and substantial heterogeneity exists across studies.

## 1. Introduction

Chronic kidney disease (CKD) is a progressive condition characterized by structural or functional changes in the kidney that persist for more than 3 months, with or without deterioration of kidney function; or a glomerular filtration rate (GFR) of less than 60 mL/min/1.73 m^2^ without other signs of kidney disease [1]. Some risk factors for CKD are diabetes, high blood pressure, heart disease, smoking, and obesity [1]. Common markers of kidney damage include high levels of albuminuria and proteinuria, alterations in the urinary sediment, electrolyte alterations and other alterations of tubular origin, histological structural alterations, and structural alterations in imaging tests [2].

Kidney disease is generally classified into five categories or grades based on GFR: Grade 1, GFR > 90 mL/min/1.73 m^2^, >3 months; Grade 2: GFR 60 to 89 mL/min/1.73 m^2^, >3 months; Grade 3a: GFR of 45–59 mL/min/1.73 m^2^, >3 months; Grade 3b: GFR 30 to 44 mL/min/1.73 m^2^, >3 months; Grade 4: 15 to 29 mL/min/1.73 m^2^, >3 months; Grade 5: GFR < 15 mL/min/1.73 m^2^, >3 months [2]. The diagnosis is made through urine and blood tests, which can sometimes be complemented with an ultrasound, scan, magnetic resonance image or even biopsy [3]. However, CKD does not usually present symptoms until it is advanced, being an underdiagnosed pathology due to the lack of apparent symptoms in the initial stage [1]. CKD is an important public health problem [1,3]. The prevalence of CKD varies between 10 and 13.4% of the world population [1,4]. According to data from the EPIRCE study [2], it affects approximately 10% of the Spanish adult population and more than 20% of those over 60 years of age, causing 1.2 million deaths a year worldwide. According to the World Health Organization (WHO), the figure will reach 5–10 million annually [1], and it is estimated that in the coming years it may be the 4th leading cause of mortality worldwide [4]. CKD represents a growing global public health challenge not only due to its increasing prevalence but also because of its systemic inflammatory implications [5], which require collaboration across medical and dental disciplines [6]. International evidence emphasizes the need for integrated care strategies in CKD management, where nephrology nurses play a critical role in patient monitoring, systemic complication prevention, and interdisciplinary coordination [7].

On the other hand, chronic oral inflammatory diseases, such as AP and PD, are highly prevalent worldwide in the general population [8,9]. However, reported prevalence figures for AP and PD should be interpreted cautiously, as they vary considerably depending on diagnostic thresholds and imaging techniques.

Apical periodontitis (AP) is the inflammation and destruction of the apical periodontium that is of pulpal origin [10] and is generally caused by the evolution without treatment of a pulp lesion caused by caries [11]. AP can be acute (symptomatic) or chronic (asymptomatic) [10]. Chronic AP is a highly prevalent pathology; according to [8], 52% of the world’s population is considered to have at least one tooth with AP. Radiographically, chronic AP is characterized by a radiolucent lesion around the apex of the affected tooth caused by the destruction of the periapical bone [12]. The treatment of AP consists of eradicating, or significantly reducing, the microbial load of the root canal and preventing reinfection by obturating the root canal system [13].

Periodontitis (PD) [14] is a chronic inflammatory condition affecting the supporting tissues of the teeth and is primarily induced by bacterial biofilm accumulation leading to progressive destruction of the periodontal ligament and alveolar bone [15]. The pathogenesis of PD is multifactorial, involving microbial dysbiosis and an exacerbated host immune-inflammatory response that contributes to tissue breakdown [16].

Oral inflammatory/infectious processes, such as AP and PD, can have systemic repercussions [11,17]. Several studies have found an association between the prevalence of AP and several systemic diseases such as diabetes, cardiovascular diseases, and smoking habits [11,18]. Like AP, PD has been associated with systemic conditions, suggesting a bidirectional relationship with diseases such as diabetes mellitus and cardiovascular disease [19,20].

The underlying mechanisms linking AP and PD with systemic diseases include persistent low-grade inflammation, bacteremia, and the dissemination of pro-inflammatory mediators such as interleukins, tumor necrosis factor-alpha, and C-reactive protein [11,21]. Moreover, both PD and AP may share similar pathogenic pathways involving microbial challenge and host response, which could potentiate systemic inflammatory burden, potentially contributing to or reflecting systemic dysregulation [22,23]. Therefore, understanding the interplay between these oral inflammatory conditions and systemic health is essential for comprehensive patient management. However, studies investigating the possible association of AP and PD with chronic renal failure have found conflicting results [11,24,25,26].

The objective of this systematic review was to analyze the existing evidence regarding the relationship between the prevalence of chronic oral infections, including AP and PD, and CKD. The research question was as follows: In adult subjects, does the presence of oral infections, such as AP and PD, compared with their absence, affect the prevalence of chronic kidney disease? The null hypothesis tested was there is no significant association between the prevalence of chronic oral inflammatory diseases and CKD.

## 2. Materials and Methods

This systematic review of observational studies is reported in accordance with PRISMA guidelines [27]. PROSPERO registration code: CRD420251167323.

### 2.1. Review Question

The review question, structured according to the PECO method (population, exposure, comparison, outcome)—i.e., In adult subjects (P), does the presence of chronic kidney disease (E), compared with the absence of CKD (C), affect the prevalence of chronic oral inflammatory disease, AP or PD (O)?—was the reference for the inclusion and exclusion criteria.

### 2.2. Eligibility Criteria

The inclusion criteria were as follows: (a) epidemiological studies published until September 25, 2025; (b) studies comparing chronic kidney disease patients with healthy control subjects; and (c) studies assessing the prevalence of AP or PD, both in CKD patients and in control healthy subjects.

The exclusion criteria were as follows: (1) in vitro or animal studies; (2) case series; (3) studies reporting data only from CKD patients, without controls; and (4) studies not reporting the prevalence of oral infections.

### 2.3. Search Strategy and Information Sources

The terms used for the search were as follow: (“Periapical Periodontitis”[MeSH Terms] OR “apical periodontitis”[All Fields] OR “periapical lesion*”[All Fields] OR “periapical disease*”[All Fields] OR (“Periodontal Diseases”[MeSH Terms] OR “periodontitis”[All Fields] OR “chronic periodontitis”[All Fields] OR “periodontal disease*”[All Fields] OR “gum disease*”[All Fields])) AND (“chronic kidney disease*”[All Fields] OR “CKD”[All Fields] OR “chronic renal disease*”[All Fields] OR “chronic renal insufficiency”[All Fields]) AND (“association”[All Fields] OR “relationship”[All Fields] OR “link”[All Fields] OR “correlation”[All Fields] OR “risk factor*”[All Fields] OR “association study”[All Fields]). The same search string was used in all databases, with minor syntax adjustments when required by database-specific interfaces. The exact search strings used for each database are shown in a Supplementary Table S1. The last day of the search was 25 September 2025 for all databases.

The investigators performed independent electronic searches of the PubMed/MEDLINE, Web of Science, Scopus, and EMBASE databases. Articles were selected by three authors individually (J.J.S-E, L.L-S., and J.L-L.). References of the included articles were also hand searched. All three authors examined the title and abstract of the articles found to determine their eligibility and then analyzed the full text of those that appeared to meet the criteria to finally make the decision to include or exclude them. Disagreements about eligibility were resolved by discussion and consensus. Full texts of all selected studies were then obtained. In addition, hand searches of some journals, article reference lists, and gray literature were conducted using the ProQuest and OpenGrey databases.

### 2.4. Data Extraction

Two of the authors (L.L-S., C.S.-R.) were responsible for data extraction, while three reviewers (J.J.S-E., J.J.S-S, and J.L-L.) verified the tabulated data to ensure the absence of typo errors and carried out the analysis of the articles; the articles in disagreement were discussed. To analyze and synthesize the data, the following details were extracted from the studies: author and year of publication, study design, sample size, objective, and results. Pooled estimates from the studies were analyzed using a random-effects model meta-analysis.

### 2.5. Data Synthesis and Analysis

The outcome variables analyzed were the prevalence of AP and PD among patients with CKD and healthy control subjects. In each selected study, the odds ratio (OR) was calculated with its 95% confidence interval (CI), aiming to measure the effect of the relationship between CKD and the prevalence of oral infections.

To determine the overall OR and its 95% CI for the prevalence of oral infections, AP or PD, a random-effects meta-analysis was performed using RevMan 5.4 software [28]. Forest plots were produced to graphically represent the odds ratio of the association between oral infections and CKD. Subgroup analysis was performed for AP and PD. To determine the heterogeneity among trials, the Tau^2^ and the Higgins I^2^ tests were employed, considering that substantial heterogeneity is considered if the I^2^ test result is higher than 50% [29]. The level of significance was applied to *p* = 0.05.

### 2.6. Risk of Bias Assessment

Each selected study was evaluated for inner methodological risk of bias independently by two authors (J.J.S.-E. and J.L.-L.). In the case of disagreements over the risk of bias, it was discussed until a consensus was achieved. The risk of bias of the included studies was evaluated using the Newcastle–Ottawa Scale adapted for cross-sectional studies [30,31]. This scale was adapted to the outcome of interest, classifying the items into three domains: sample selection, comparability, and outcome. They were given a point (*) depending on whether the aspects required were present or missing. The following criteria were used to evaluate each section:

Domain “Sample selection” (maximum = 3 points).

- Representativeness of the sample: random sampling: two points; non-random sampling or selected group of patients: one point; no explanation of the sampling selection: no points.
- Sample size: the methods for sample size calculation are provided, or the entire population was enlisted (with loss rate ≤ 20%): one point; sample size calculation not provided: no points.

Domain “Comparability” (maximum = 3 points).

- If the study controlled for or adjusted for the presence of diabetes mellitus (a causal factor in CKD and periodontitis): one point.
- If the study controlled for age, another confounding factor (due to its influence on both conditions): one point; not controlling: no points.
- If the study controlled for another factor, such as sex, smoking habits, socioeconomic level, hypertension, oral hygiene, or duration of dialysis or stage of CKD: one point; not controlling: no points.

Domain “Outcome” (maximum = 4 points).

- If the diagnosis of AP or PD was based on valid and standardized clinical or radiographic criteria: one point; if the diagnosis was subjective, without defined criteria, or based solely on clinical observation without calibration: no points.
- If the number of observers was two or more: one point; if only one: no points.
- If the examiners were calibrated or inter- and intra-examiner reliability was reported, performing examiner blinding with respect to the group (CKD vs. control): one point; if there was no calibration and inter- and intra-examiner reliability was not reported: no points.
- If the statistical analysis was appropriate to the design (e.g., calculation of OR and 95% CI, use of logistic regression or chi-square as appropriate) and the complete results were reported for all included participants, without data loss or unjustified exclusions: one point; if the analysis was descriptive or incomplete, or the results were not reported by the group: no points.

The lowest possible risk of bias was scored with 10 points. Studies with scores from 0 to 4 points were considered to be at a high risk of bias, those with scores between 5 and 7 points were considered to be at moderate risk of bias, and finally, studies with scores between 8 and 10 points were considered to be at low risk of bias.

### 2.7. Grading of Recommendations Assessment, Development and Evaluation

The Grades of Recommendation, Assessment, Development and Evaluation (GRADE) approach [32] was used for the assessment of the overall quality of the evidence. This procedure defines an initial level of certainty according to the design of the included studies and, subsequently, analyzes different domains such as the risk of bias, inconsistency, indirectness, imprecision, publication bias, dose-response gradient, confounding factors, or the magnitude of the effect to conclude at a final level of certainty. A moderate or high level of evidence indicates that confidence in the result is medium or high. Conversely, a low or extremely low level of evidence indicates that confidence in the result is limited or extremely weak, respectively [33].

## 3. Results

**Figure 1:**
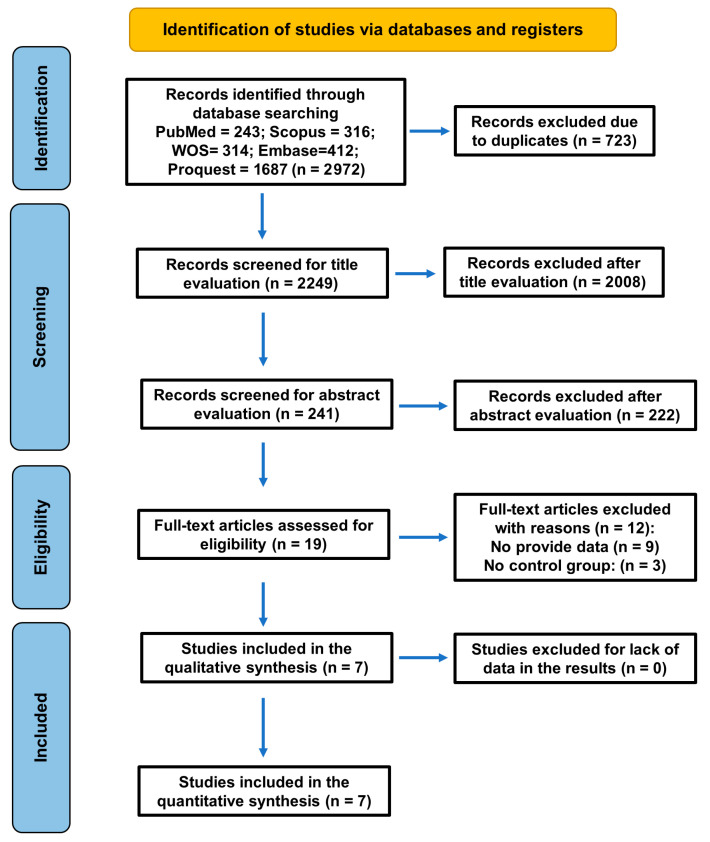
Flowchart of the search strategy following the PRISMA 2020 guidelines for systematic reviews and meta-analyses.

The baseline search recovered 2972 titles. After the removal of duplicates (n = 723 excluded) and the screening for title evaluation (2008 excluded), 241 articles remained, of which 222 were excluded after reading the abstracts for being unrelated to the topic, and 19 were selected for full text. After full-text reading, twelve articles were excluded for the following reasons (Table 1): in two there was an absence of a control group [34,35], and nine did not provide the necessary data [24,25,36,37,38,39,40,41,42]. Finally, seven studies fulfilled the inclusion criteria and were selected for systematic review and meta-analysis (Figure 1).

Finally, seven studies fulfilled the inclusion criteria and were selected for systematic review and meta-analysis (Figure 1).

### 3.1. Characteristics of the Included Studies

The characteristics of the seven studies selected and included for systematic review and meta-analysis are shown in Table 2. All studies were published between 2011 and 2024, representing diverse geographical regions—Asia (India, Nepal, Iran, Saudi Arabia, Nigeria) and North America (United States).

One study was cross-sectional and population-based [43], two were matched case-control studies [22,44,45], and the remaining three were unmatched case-control studies [26,46,47]. Two studies analyzed the association between CKD and AP [26,44], and five studies analyzed the association between CKD and PD [43,45,46,47,48].

**Table 2:** Studies on the prevalence of chronic oral infections, namely periodontitis, and apical periodontitis in chronic kidney disease (CKD) patients and control subjects included in the systematic review and meta-analysis.

Author, Year	Study Design	Sample	Oral Infection	Diagnostic Criteria for Oral Infections	Main Outcome
Ioannidou & Swede, (2011) United States [43]	Cross-sectional (NHANES III)	706 CKD patients 11375 controls	Periodontitis	CDC/AAP criteria	PD significantly more frequent in CKD patients OR = 1.8; 95%IC = 1.5–2.3 *p* = 0.000
Oyetola et al. (2015) Nigeria [46]	Case-control	90 CKD patients 90 controls	Periodontitis	Periodontitis was diagnosed when PPD > 3 mm	Periodontitis significantly more frequent in CKD patients *p* < 0.01
Khalighinejad et al. (2017) Iran [44]	Matched case-control	40 ESRD patients 40 controls	Apical periodontitis	≥1 tooth with AP (digital panoramic radiographs and pulp test)	AP significantly more frequent in ESRD patients OR = 3.95; 95% IC = 1.54–6.32 *p* < 0.05
Munagala et al. (2022) India [45]	Case-control	75 CKD hemodialysis patients 75 controls	Periodontitis	CDC/AAP criteria	Periodontitis significantly more frequent in CKD patients *p* < 0.001
Lamba et al. (2023) India [26]	Case-control	105 CKD patients 105 controls	Apical periodontitis	≥1 tooth with AP (CBCT)	AP significantly more frequent in CKD patients OR = 3.95; 95%IC = 2.09–7.45 *p* < 0.001
Joshi et al. (2024) Nepal [47]	Case-control	54 CKD dialysis patients 54 controls	Periodontitis	CDC/AAP criteria	O R= 5.3; 95%CI = 1.9–14.7 *p* = 0.000
Rani et al. (2024) India [22]	Matched case-control	165 ESRD patients 165 controls	Periodontitis	Modified CPI and WHO criteria LOA > 3 mm/PPD > 4 mm	Periodontitis significantly more frequent in ESRD patients *p* < 0.001

AAP: American Academy of Periodontology; AP: apical periodontitis; BOP: bleeding on probing; CAL: clinical attachment level; CBCT: cone beam computed tomography; CDC: Centers for Disease Control; CPI: Community Periodontal Index; ESRD: end-stage renal disease; LOA: loss of attachment; PI: plaque index; PPD: periodontal probing depth; WHO: World Health Organization.

Regarding the diagnosis of oral infections, the two studies that analyzed the prevalence of AP used the criterion that the patient had at least one periapical lesion radiographically diagnosed using CBCT [26] or a digital panoramic radiograph [44]. Regarding periodontitis, it was diagnosed using the criteria of CDC/AAP [49] in three of the included studies [43,45,47], and periodontal probing depth was used in the other two studies [22,46]. The results of the seven studies showed a significantly higher prevalence of oral infection in patients with CKD.

An evidence table was created with data from the included studies (Table 3). The number of patients with oral infection, AP or PD, in both the CKD patients and control subjects was the key data extracted in each of the studies. Then, the prevalence of oral infection in both groups was calculated, as well as the corresponding OR for each study.

A total of 13,139 individuals were analyzed in the seven included articles [22,26,43,44,45,46,47], of which 1235 had CKD, 145 were diagnosed with AP, and 1090 with PD. The remaining 11,904 were healthy controls without CKD.

The patients with CKD showed a prevalence of oral infection (41.9%) significantly higher than healthy controls (10.2%). The OR values calculated for the seven included studies ranged from 1.79 (95%CI = 1.44–2.23) [43] to 38.50 (95%CI: 15.18–97.64).

### 3.2. Meta-Analysis of the Prevalence of Oral Infections in CKD Patients

**Figure 2:**
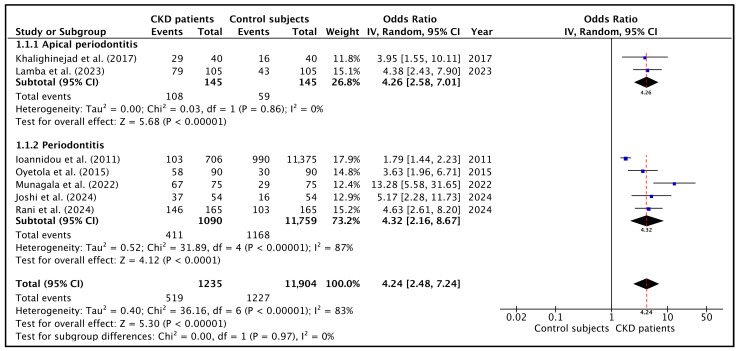
Forest plot of the OR and its 95% confidence interval comparing the prevalence of oral infections in CKD patients and healthy control subjects. **Top**: the estimate was based on data from the two studies on the prevalence of apical periodontitis. **Middle**: the estimate was based on data from the five studies on the prevalence of periodontitis. **Bottom**: the estimate was based on data from the seven selected studies on oral infections. The red dotted lines indicate the overall OR in each forest plot [22,26,43,44,45,46,47].

The data shown in Table 3 were used to perform the meta-analysis. The forest plot is shown in Figure 2. When pooling all studies for quantitative synthesis (Figure 2, bottom), the overall OR for the association between CKD and the presence of oral infections was 4.9 (95% CI: 2.6–9.5; *p* < 0.001), indicating that individuals with CKD are approximately five times more likely to present oral inflammatory pathology compared with healthy controls. This suggest a strong association between chronic kidney disease (CKD) and the prevalence of chronic oral infections such as AP and PD.

Heterogeneity was high (I^2^ = 83.0%; *p* < 0.00001), mainly reflecting differences in diagnostic criteria and the proportion of dialysis versus non-dialysis participants across the studies. Although heterogeneity among studies was moderate to high, the overall direction of effect was uniform across all analyses, with CKD patients consistently exhibiting a higher prevalence of oral disease than non-CKD controls.

Regarding subgroup analysis, the pooled meta-analysis including the two studies [26,44] reporting the prevalence of AP in CKD patients (Figure 2, top) demonstrated that these patients were approximately four times more likely to present with AP than controls (OR = 4.3; 95% CI = 2.6–7.0; *p* < 0.01), indicating a consistent and significant association. Heterogeneity was very low (I^2^ = 0%; *p* = 0.86).

On the other hand, the pooled OR calculated with the studies on PD demonstrated that CKD individuals were more than five times more likely to present with PD (OR = 4.3; 95% CI = 2.2–8.7; *p* = 0.00001) (Figure 2, middle). Heterogeneity was very high (I^2^ = 87%; *p* < 0.0001), reflecting differences in periodontal case definitions, disease severity thresholds, and the inclusion of dialysis versus non-dialysis CKD populations.

### 3.3. Sensitivity and Publication Bias

Sensitivity analysis excluding the study with the most extreme effect size [45] reduced the pooled OR from 4.9 to 3.5, but the association remained statistically significant (*p* < 0.001). Separate analyses excluding studies without standardized definitions of periodontitis and those based exclusively on dialysis populations indicated that the observed association was not due to any particular study or methodological variability between subgroups.

**Figure 3:**
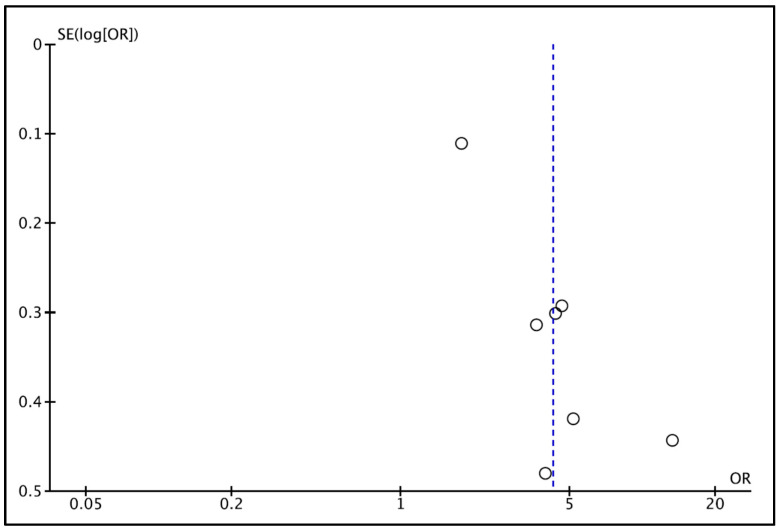
Funnel plot for estimates in the meta-analysis of the prevalence of oral infections in CKD patients and healthy control patients. The vertical blue line shows the pooled effect estimate from the random-effects model. Studies with higher power levels and lower standard error are placed toward the top. Studies with lower power levels are placed toward the bottom.

Regarding publication bias, the funnel plot (Figure 3) visually assessed potential publication bias among the seven studies included in the meta-analysis. The distribution of studies appeared slightly asymmetrical, with smaller studies showing a tendency toward higher odds ratios [45], while larger population-based studies [43] clustered near the pooled effect estimate. Despite this mild asymmetry, the overall pattern did not suggest substantial publication bias. The absence of extreme outliers—other than in one small study with a large effect size—indicated that the observed heterogeneity was primarily due to clinical and methodological variability rather than selective reporting. Visual inspection therefore supports the robustness of the pooled estimate, which remained significant in sensitivity analyses after excluding small, high-effect studies. No evidence of systematic bias was detected, consistent with the Egger’s regression test (*p* = 0.12). Given the limited number of studies, the analyses were underpowered to detect small-study effects; therefore, no firm conclusions regarding publication bias can be drawn.

### 3.4. Risk of Bias Assessment

According to the Newcastle–Ottawa Scale, risk of bias was evaluated for each study (Table 4). One of the included studies was classified as low risk of bias [43], four were classified as having moderate risk of bias [22,26,44,45], and the remaining two [46,47] were assessed as having a high risk of bias.

The main source of potential bias was confounding, particularly inadequate adjustment for diabetes, smoking, or socioeconomic status—key variables that can influence both CKD and oral inflammatory diseases.

The cross-sectional population-based study [43] achieved the lowest overall risk, being classified as low risk in most domains due to comprehensive adjustment and standardized diagnostic methods. In contrast, two studies [46,47] were rated as serious risk primarily because of unmeasured confounders and convenience sampling.

### 3.5. GRADE Assessment of Quality of Evidence

**Figure 4:**
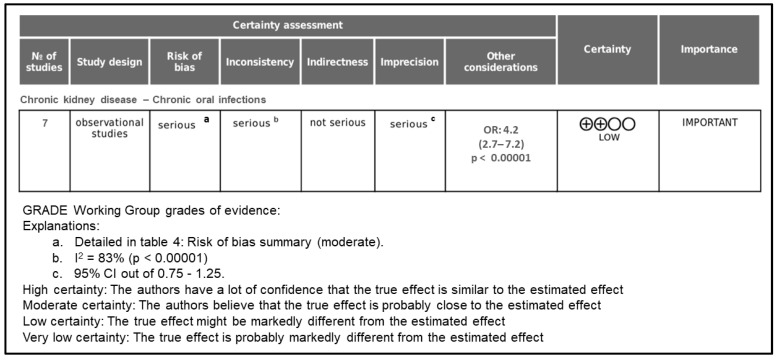
Grade assessment of evidence certainty.

The certainty of the evidence was evaluated according to the GRADE (Grading of Recommendations Assessment, Development and Evaluation) approach, which considers five domains: risk of bias, inconsistency, indirectness, imprecision, and publication bias. As illustrated in Figure 4, the overall certainty of the evidence supporting the association between chronic kidney disease (CKD) and oral inflammatory diseases (periodontitis and apical periodontitis) was rated as low.

The initial certainty level started as low because all included studies were observational. The evidence was upgraded due to the large magnitude of effect observed in the meta-analysis (OR = 4.2; 95% CI = 2.5–7.2; *p* < 0.00001), as well as the consistency of results across different populations and study designs.

However, the overall certainty was downgraded in three domains:

- Risk of bias was rated as serious because most included studies presented moderate methodological limitations, such as incomplete control for major confounders (e.g., diabetes, smoking, socioeconomic status) and lack of examiner calibration in the diagnosis of oral diseases.
- Inconsistency was downgraded due to the high level of heterogeneity among studies (I^2^ = 83.0%), which likely reflects variations in diagnostic criteria, CKD stages, and sample characteristics.
- Imprecision was considered serious because of the limited number of studies (n = 7) and wide confidence intervals in some pooled estimates.

By contrast, indirectness and publication bias were not considered serious concerns. All included studies directly compared CKD patients with healthy controls using validated diagnostic criteria, and no significant asymmetry was observed in the funnel plot or Egger’s regression test (*p* = 0.12).

Taken together, these findings indicate that while the association between CKD and oral inflammatory diseases appears consistent and biologically plausible, the confidence in the estimated effect remains limited. Consequently, the certainty of the evidence was classified as low, suggesting that the true effect may differ substantially from the observed estimate.

## 4. Discussion

The aim of this systematic review and meta-analysis was to assess the relationship between CKD and chronic oral inflammatory conditions, specifically PD and AP. The analysis included seven observational studies published between 2011 and 2024, encompassing over 13,000 individuals from diverse geographic regions. The pooled results revealed that CKD patients were approximately five times more likely to present with oral inflammatory pathologies compared to healthy controls (4.2; 95% CI: 2.5–7.2; *p* < 0.00001). Subgroup analysis demonstrated similar stronger association for PD (OR = 4.3) and for AP (OR = 4.3). These findings suggest a substantial and clinically meaningful relationship between renal dysfunction and the prevalence of chronic inflammatory diseases.

### 4.1. Interpretation of the Main Findings

The results of this meta-analysis suggest a positive association between chronic kidney disease (CKD) and chronic oral inflammatory pathologies, including both PD and AP. However, the strength and consistency of this relationship vary across studies. Given the high heterogeneity and overall low certainty of evidence according to GRADE, these findings should be interpreted with caution.

This association likely reflects a complex interplay of systemic inflammation, immune dysregulation, and altered bone metabolism that characterizes CKD and predisposes affected individuals to chronic oral infections. The finding that CKD patients have approximately fivefold higher odds of presenting oral inflammatory disease suggests that renal impairment significantly amplifies the susceptibility to oral tissue breakdown and delayed healing.

From a pathophysiological standpoint, the observed relationship could be interpreted as bidirectional. CKD promotes a chronic inflammatory milieu, oxidative stress, and uremic toxicity, which collectively impair host defense and periodontal and periapical repair mechanisms, and, conversely, AP and PD act as persistent sources of systemic inflammation, potentially aggravating renal dysfunction through elevated circulating levels of pro-inflammatory cytokines such as IL-6, TNF-α, and CRP [47,50]. This reciprocal influence may help explain the worsening of both oral and renal conditions over time in affected patients. However, although biological plausibility supports a bidirectional relationship between oral and systemic inflammation, the cross-sectional and case-control designs included in this review preclude the inference of causal direction.

The stronger associations observed in studies involving dialysis populations [22,45,47] suggest that disease severity plays a critical role, with advanced CKD stages conferring higher vulnerability to oral infection. In contrast, the more modest association detected in population-based research [43] may reflect inclusion of earlier CKD stages and better systemic control of confounding factors such as diabetes and hypertension.

Overall, these results indicate that oral inflammatory disease should be considered a comorbid condition of CKD, and that oral health status may serve as a marker of systemic inflammatory burden in this population. The magnitude and consistency of the pooled effect across diverse geographic and methodological contexts reinforces the biological plausibility and clinical relevance of this relationship.

Importantly, these findings highlight the potential benefits of incorporating periodontal and endodontic screening into nephrology care protocols, as early detection and management of oral infection could mitigate systemic inflammation and improve patient outcomes.

### 4.2. Methodological Considerations

The methodological framework of this systematic review and meta-analysis was designed to maximize reliability and minimize bias in synthesizing evidence on the association between CKD and oral inflammatory diseases. The study adhered to PRISMA guidelines for systematic reviews [27] and incorporated only observational studies that directly compared CKD patients with healthy controls, ensuring the clinical comparability of groups.

Data extraction was conducted independently by two of the authors, and three reviewers verified the tabulated data to ensure the absence of typo errors. A random-effects model was used to calculate pooled odds ratios, acknowledging the inherent heterogeneity in study design, population characteristics, and diagnostic criteria. This approach allowed for generalization beyond individual samples while maintaining statistical conservatism.

Heterogeneity was high (I^2^ = 83.0%) but acceptable given the diversity of methodologies, and sensitivity analyses confirmed the robustness of the findings; excluding high-effect studies [45] did not substantially alter the overall association.

Quality appraisal was performed using the Newcastle–Ottawa Scale adapted for cross-sectional studies [28,29]. Most studies were rated as having a low or moderate risk of bias, and only two were classified as having a high risk of bias. Similarly, most studies applied standardized clinical or radiographic criteria, such as the CDC/AAP classification for PD and CBCT or periapical imaging for periapical lesions, ensuring low risk of misclassification. Regarding the case definitions for periodontitis, CDC/AAP criteria were established jointly by the Centers for Disease Control and Prevention (CDC) and the American Academy of Periodontology (AAP) [49]. According to these criteria, periodontitis is classified based on clinical attachment loss (CAL) and probing pocket depth (PPD) measured at multiple sites, allowing differentiation between mild, moderate, and severe disease. Despite these variations, the diagnostic assessment of both PD and AP was generally reliable and consistent. Moreover, CKD diagnosis in all studies was based on objective clinical or laboratory parameters (eGFR thresholds, dialysis status, or medical records), minimizing exposure bias.

In summary, the methodological design and analytical rigor of this review provide confidence that the observed association between CKD and oral chronic inflammatory diseases reflects a true biological and clinical relationship, rather than an artifact of study design or measurement error.

### 4.3. Comparison with Other Studies

The findings of this meta-analysis are in agreement with a growing body of literature highlighting the close interconnection between chronic kidney disease (CKD) and oral inflammatory pathologies, particularly PD and AP. Although the present review included seven eligible studies, similar investigations not incorporated in this analysis have consistently supported the same direction of association. For instance, a large multicenter observational study [51] reported that over 60% of hemodialysis patients exhibited moderate to severe periodontal disease, confirming the elevated burden of oral inflammation among CKD populations. Their conclusions align with our pooled estimates, suggesting that systemic inflammation and reduced immune competence in CKD patients predispose them to periodontal breakdown. Similarly, Grubbs et al., (2016) [52] demonstrated that periodontal inflammation correlates positively with serum levels of C-reactive protein (CRP) and interleukin-6 (IL-6) in individuals with impaired renal function, further strengthening the biological plausibility of our findings.

Additional evidence from interventional and mechanistic studies supports the bidirectional link between CKD and oral disease. For example, it has been shown that non-surgical periodontal therapy in hemodialysis patients significantly reduced systemic inflammatory markers and improved endothelial function, indicating that oral infection contributes to systemic inflammatory load [53]. Likewise, it has been observed that treatment of chronic periodontitis resulted in decreased serum creatinine levels and improved estimated glomerular filtration rate (eGFR), suggesting that oral inflammation may exacerbate renal dysfunction through systemic cytokine release [54,55].

In contrast, some earlier population-based analyses reported weaker associations or non-significant findings after full adjustment for confounders [56,57]. These discrepancies may be explained by differences in CKD definition, periodontal diagnostic thresholds, and the inclusion of early-stage renal impairment rather than advanced disease. Nevertheless, even these studies observed consistent trends toward higher inflammatory burden and poorer oral health among CKD participants.

Taken together, the convergence of evidence from cross-sectional, longitudinal, and interventional studies strongly supports the biological and clinical interdependence between CKD and oral inflammation. However, although biologically plausible, the observed association should be interpreted as correlational rather than causal.

The present meta-analysis strengthens this evidence base by quantifying the association across diverse populations and diagnostic approaches, confirming that oral chronic inflammatory diseases could be not merely a local condition but a systemic manifestation intertwined with renal health.

### 4.4. Study Limitations

Despite the methodological rigor applied in this systematic review and meta-analysis, several limitations should be acknowledged when interpreting the findings. First, the evidence synthesized in this study is derived exclusively from observational designs, which preclude causal inference. While strong associations were observed between CKD and both PD and AP, the temporal sequence between renal dysfunction and the onset or progression of oral inflammation cannot be definitively established. Future prospective cohort studies and interventional trials are needed to clarify whether the relationship is unidirectional or bidirectional in nature.

Second, the heterogeneity among studies (high, I^2^ = 83%) reflects variability in diagnostic criteria, population characteristics, and disease staging. Differences in case definitions of periodontitis (CDC/AAP, CPI, or clinical categories) and in CKD classification (early vs. end-stage disease, dialysis vs. non-dialysis) likely contributed to variations in effect size. Nevertheless, the consistent direction of association across all analyses supports the robustness of the findings.

Third, most of the included studies were hospital-based and relatively small, which may limit the generalizability of results to broader CKD populations. Such studies often rely on convenience sampling and may over-represent patients with advanced disease or comorbidities. In contrast, population-based studies [34] provided more representative samples but fewer data on disease severity and treatment history.

Residual confounding remains an important limitation. Although some studies adjusted for major factors such as age, sex, diabetes, and smoking, others lacked multivariate analysis, raising the possibility that unmeasured variables, such as oral hygiene practices, nutrition, medication use (e.g., immunosuppressant), or socioeconomic status, may partially explain the observed associations. Additionally, biological markers of systemic inflammation (e.g., CRP, IL-6, TNF-α) were not uniformly assessed, limiting the ability to explore mechanistic pathways linking oral and renal inflammation.

A further limitation concerns the diagnostic assessment of periodontal and apical pathologies. While several studies used standardized radiographic or clinical criteria, others relied on examiner judgment without calibration, introducing potential measurement bias. Similarly, differences in imaging techniques (CBCT vs. periapical radiographs) could affect the sensitivity of apical lesion detection.

A limitation of this study, regarding the meta-analysis, is that the NHANES-based study contributed unweighted 2 × 2 data, although NHANES uses a complex sampling design with survey weights. This may introduce some bias relative to the original weighted estimates, and the corresponding results should therefore be interpreted with caution.

Finally, although no significant publication bias was identified through funnel plot and Egger’s regression analyses, the small number of eligible studies (n = 7) limits the statistical power of these methods. It is therefore possible that smaller studies with null results remain unpublished.

Despite these limitations, the convergence of findings across independent research groups and populations strengthens the confidence in the observed association. Together, the evidence supports a meaningful link between CKD and oral inflammatory diseases, warranting further investigation through longitudinal, multicenter, and mechanistic studies designed to establish causality and evaluate the impact of oral health interventions on renal outcomes.

### 4.5. Clinical Implications

The findings of this meta-analysis underscore the critical need for integrated medical–dental management in patients with chronic kidney disease (CKD). Given the strong association between CKD and oral inflammatory diseases, routine periodontal and endodontic assessment should be incorporated into nephrology care protocols. Early detection and control of oral infection may help to reduce systemic inflammation, improve metabolic stability, and potentially slow renal deterioration.

The clinical implications of the present findings extend beyond traditional biomedical perspectives, highlighting the need for an integrated, multidimensional, and multidisciplinary approach to the management of patients with chronic kidney disease (CKD). Given the demonstrated association between CKD and chronic oral infections, including periodontitis and apical periodontitis, it is essential to incorporate oral health assessment into routine nephrology care pathways as part of comprehensive risk reduction strategies [51]. From an assistive practice standpoint, nephrology nurses and allied health professionals play a central role in early identification of oral health problems, patient education, reinforcement of hygiene behaviors, and coordination of interprofessional referrals, particularly for high-risk populations such as hemodialysis patients [58]. Modern models of chronic disease management emphasize collaborative care involving nephrologists, dental specialists, primary care providers, and nursing teams to reduce the systemic inflammatory burden and improve overall quality of life in CKD patients. Consequently, our findings support the growing international call for integrated medical–dental guidelines and the development of interprofessional care protocols that bridge nephrology and oral medicine across both clinical and community settings.

## 5. Conclusions

This systematic review and meta-analysis highlight a potential link between chronic oral inflammatory diseases, including both AP and PD, and chronic kidney disease (CKD), which warrants further investigation through well-designed longitudinal and interventional studies. However, the certainty of the evidence is low, and substantial heterogeneity exists across studies. The observational nature of the available data precludes any inference of causality or directionality. While biological plausibility supports potential links between oral and systemic inflammation, these findings should be interpreted with caution. Future longitudinal and interventional studies using standardized diagnostic criteria and comprehensive adjustment for confounding factors are needed to clarify the mechanisms underlying these associations and to determine their clinical implications.

## Figures and Tables

**Table 1:** Excluded studies and their reasons for exclusion.

Reasons	Excluded Studies
Absence of control group	Buhlin et al., 2007 [34] Katz et al., 2021 [35] Palmeira et al., 2024 [23]
Lack of necessary data about the prevalence of periodontitis	Thorman et al., 2009 [39] Ma et al., 2016 [42] Altamini et al., 2018 [25] Dembowska et al., 2022 [24]
Lack of necessary data about the prevalence of apical periodontitis	Sobrado et al., 2007 [36] Bayraktar et al., 2007 [37] Garcez et al., 2009 [38] Tadakamadla et al., 2013 [40] Niedzielska et al., 2014 [41]

**Table 3:** Studies on the prevalence of chronic oral infections, namely periodontitis and apical periodontitis, in chronic kidney disease (CKD) patients. Results extracted and compiled with descriptive statistics and odds ratios calculated.

Author, Year, Country	Oral Infection	No. Subjects	CKD Patients		Control Subjects		Odds Ratio
			AP or PD/Total	Prevalence CKD (%)	AP or PD/Total	Prevalence Controls (%)	
Ioannidou & Swede (2011) United States [43]	PD	12,081	103/706	14.6	990/11,375	8.7	OR = 1.79 95% CI = 1.44–2.23 *p* = 0.000
Oyetola et al. (2015) Nigeria [46]	PD	180	58/90	64.4	30/90	33.3	OR = 3.62 95% CI = 1.96–6.71 *p* = 0.000
Khalighinejad et al. (2017) Iran [44]	AP	80	29/40	73	16/40	40	OR = 3.95 95% CI = 1.54–6.32 *p* < 0.05
Munagala et al. (2022) India [45]	PD	150	67/75	89.3	29/75	38.7	OR = 13.28 95% CI = 5.58–31.64 *p* = 0.0000
Lamba et al. (2023) India [26]	AP	210	79/105	75.2	43/105	41.0	OR = 3.95 95% CI = 2.09–7.45 *p* < 0.001
Joshi et al. (2024) Nepal [47]	PD	108	37/54	68.5	16/54	29.6	OR = 5.17 95% CI = 2.28–11.73 *p* = 0.000
Rani et al. (2024) India [22]	PD	330	147/165	88.5	103/165	62.4	OR = 4.63 95% CI = 2.61–8.20 *p* = 0.000
OVERALL		13,139	518/1235	41.9	1227/11,904	10.3	

AP: apical periodontitis; CKD: chronic kidney disease; PD: periodontitis.

**Table 4:** Assessment of risk of bias according to Newcastle–Ottawa Scale adapted for cross-sectional studies.

Author 2011.	Sample Selection		Comparability			Outcome				Risk of Bias
	Sample Representativeness	Sample Size	Control for Diabetes	Control for Age	Control for Other Confounding Factors	Diagnostic Criteria	No. Observers	Calibration	Statistical Analysis	
Ioannidou & Swede (2011) [43]	**	*	*	*	*	*	*	*	*	10/10 Low
Oyetola et al. (2015) [46]	*								*	2/10 High
Khalighinejad et al. (2017) [44]	*			*	*	*	*		*	6/10 Moderate
Munagala et al. (2022) [45]	*			*	*	*			*	5/10 Moderate
Lamba et al. (2023) [26]	*	*			*	*	*	*	*	7/10 Moderate
Joshi et al. (2024) [47]	*			*		*			*	4 High
Rani et al. (2024) [22]	*	*		*	*	*	*		*	7 Moderate
OVERALL	8	3	1	5	5	6	4	2	7	41/70 Moderate

As specified in Mat & Met, each of the aspects assessed in each domain were given a point (*) or (**) depending on whether the required aspects were present or missing. The maximum possible score was 10 points. Studies scoring 0–4 points were considered at high risk of bias, 5–7 points moderate risk, and 8–10 points low risk.

## Data Availability

No new data were created or analyzed in this study..

## Supplementary Materials

The following supporting information can be downloaded at https://www.mdpi.com/article/10.3390/jcm14227947/s1, Table S1: Exact search strings used in each database; PRISMA checklist.
