# Supplementary material for: Chronic Kidney Disease and Chronic Oral Inflammatory Diseases: A Systematic Review and Meta-Analysis of Periodontitis and Apical Periodontitis
Source: J Clin Med. 2025 Nov 10;14(22):7947. doi: 10.3390/jcm14227947 (PMC12653069; doi:10.3390/jcm14227947)
Supplement: Supplementary file 1 [file jcm-14-07947-s001.zip › Table S1.pdf]

Table S1. Exact search strings used in each database.

Database	Exact Search String Used	Date of last search
**PubMed/MEDLINE**	("Periapical Periodontitis"[MeSH Terms] OR "apical periodontitis"[All Fields] OR "periapical lesion*"[All Fields] OR "periapical disease*"[All Fields] OR ("Periodontal Diseases"[MeSH Terms] OR "periodontitis"[All Fields] OR "chronic periodontitis"[All Fields] OR "periodontal disease*"[All Fields] OR "gum disease*"[All Fields])) AND ("chronic kidney disease*"[All Fields] OR "CKD"[All Fields] OR "chronic renal disease*"[All Fields] OR "chronic renal insufficiency"[All Fields]) AND ("association"[All Fields] OR "relationship"[All Fields] OR "link"[All Fields] OR "correlation"[All Fields] OR "risk factor*"[All Fields] OR "association study"[All Fields])	25 September 2025
**Scopus**	("apical periodontitis" OR "periapical lesion" OR "periapical disease" OR periodontitis OR "chronic periodontitis" OR "periodontal disease") AND ("chronic kidney disease" OR CKD OR "chronic renal disease") AND (association OR relationship OR link OR correlation OR "risk factor" OR "association study")	25 September 2025
**Web of Science**	("apical periodontitis" OR "periapical lesion" OR "periapical disease" OR periodontitis OR "chronic periodontitis" OR "periodontal disease") AND ("chronic kidney disease" OR CKD OR "chronic renal disease") AND (association OR relationship OR link OR correlation OR "risk factor" OR "association study")	25 September 2025
**EMBASE**	('periapical periodontitis'/exp OR 'apical periodontitis':ab,ti OR 'periapical lesion*':ab,ti OR 'periapical disease*':ab,ti OR 'periodontal disease'/exp OR 'periodontitis':ab,ti OR 'chronic periodontitis':ab,ti OR 'periodontal disease*':ab,ti OR 'gum disease*':ab,ti) AND ('chronic kidney disease'/exp OR 'chronic kidney disease*':ab,ti OR 'CKD':ab,ti OR 'chronic renal disease*':ab,ti OR 'chronic renal insufficiency':ab,ti) AND ('association':ab,ti OR 'relationship':ab,ti OR 'link':ab,ti OR 'correlation':ab,ti OR 'risk factor*':ab,ti OR 'association study':ab,ti)	25 September 2025
**ProQuest (Grey Literature)**	("Periapical Periodontitis"[MeSH Terms] OR "apical periodontitis"[All Fields] OR "periapical lesion*"[All Fields] OR "periapical disease*"[All Fields] OR ("Periodontal Diseases"[MeSH Terms] OR "periodontitis"[All Fields] OR "chronic periodontitis"[All Fields] OR "periodontal disease*"[All Fields] OR "gum disease*"[All Fields])) AND ("chronic kidney disease*"[All Fields] OR "CKD"[All Fields] OR "chronic renal disease*"[All Fields] OR "chronic renal insufficiency"[All Fields]) AND ("association"[All Fields] OR "relationship"[All Fields] OR "link"[All Fields] OR "correlation"[All Fields] OR "risk factor*"[All Fields] OR "association study"[All Fields])	25 September 2025

The same Boolean search strategy was applied across all databases, with only minimal syntax adjustments where required by database interfaces.
